# Study protocol for the ACTIVE SCHOOL study investigating two different strategies of physical activity to improve academic performance in Schoolchildren

**DOI:** 10.1186/s12887-024-04647-9

**Published:** 2024-03-09

**Authors:** Lise Sohl Jeppesen, Linn Damsgaard, Malene Norup Stolpe, Jesper Ninn Sandfeld Melcher, Jacob Wienecke, Glen Nielsen, Søren Smedegaard, Anne Husted Henriksen, Rasmus Ahmt Hansen, Charles H Hillman, Tuija H Tammelin, Geir K Resaland, Andrew Daly-Smith, Anna Bugge

**Affiliations:** 1 Department of Midwifery, Physiotherapy, Occupational Therapy and Psychomotor Therapy, University College Copenhagen, Physiotherapy, Copenhagen, Denmark; 2 Department of Nutrition, Exercise and Sports, University of Copenhagen, Copenhagen, Denmark; 3 Faculty of Teacher Education, University College Copenhagen, Copenhagen, Denmark; 4 Faculty of Teacher Education, UCL University College, Odense, Denmark; 5 Center for Cognitive & Brain Health, Department of Psychology, Department of Physical Therapy, Movement and Rehabilitation Sciences, Northeastern University, Boston, MA USA; 6 Likes, School of Health and Social Studies, Jamk University of Applied Sciences, Jyväskylä, Finland; 7 Centre for Physically Active Learning, Faculty of Education, Arts and Sports, Western Norway University of Applied Sciences, Bergen, Norway; 8 Wolfson Centre for Applied Health Research, Faculty of Health Studies, University of Bradford, Richmond Road, Bradford, BD7 IDP UK

**Keywords:** Physical activity, School-based interventions, Implementation, Embodied learning, Academic performance, Cognitive function, Physical fitness, Well-being, School motivation

## Abstract

**Background:**

Previous research has suggested that school-based physical activity (PA) interventions may have a positive impact on academic performance. However, existing literature on school-based interventions encompasses various forms of PA, spanning from vigorous intensity PA outside the academic classes to light intensity PA and movement integrated into academic learning tasks, and results on academic performance are inconclusive. ACTIVE SCHOOL will implement two different PA interventions for one school year and assess the effects on the pupils’ academic performance, with math performance as the primary outcome.

**Methods/design:**

The ACTIVE SCHOOL project consists of two phases: 1) Development phase and 2) Randomized Controlled Trial (RCT). In phase one, two interventions were developed in collaboration with school staff. The two interventions were tested in an 8-weeks feasibility study. In phase two, a RCT-study with three arms will be conducted in 9-10-year-old children for one school year. The RCT-study will be carried out in two intervention rounds during the school years 2023/2024 and 2024/2025. Schools will be randomized to one of two interventions or control;1) *Run, Jump & Fun intervention* (4 × 30 min/week of moderate-to-vigorous physical activity; 2) *Move & Learn intervention* (4 × 30 min/week focusing on embodied learning in math and Danish lessons); or 3) a control condition, consisting of normal teaching practices. Outcome measures include academic performance, PA level, cognitive functions, cardiorespiratory fitness, anthropometry, well-being and school motivation (collected before, during and after the intervention period). A process evaluation will be conducted to assess implementation.

**Discussion:**

The ACTIVE SCHOOL study will expand knowledge regarding the impact of PA on academic performance. The study will have the potential to significantly contribute to future research, as well as the scientific and educational debate on the best way to implement PA to support education and learning.

**Trial registration:**

The study was registered on the 25th of October 2022 in ClinicalTrials.gov, NCT05602948.

**Supplementary Information:**

The online version contains supplementary material available at 10.1186/s12887-024-04647-9.

## Background

Academic performance is important both in formal education and for success later in life [1]. At the individual level, academic performance is affected by several factors and underlying mechanisms, including brain structure and function, cognitive function, mental health and well-being, school motivation, and school attitude [2, 3]. Furthermore, emerging evidence suggests positive associations between physical activity (PA), aerobic fitness, and academic outcome in children and adolescents [3], and several school-based studies have found that enhancing PA in schools can improve academic performance [4–8].

The physiological mechanisms by which PA affects academic performance are representing a rapidly growing area of multidisciplinary research. Animal models provide cellular and molecular explanations for altered brain function after PA training, and imaging models on humans show how specific brain structures are affected by PA [9, 10]. In children, some studies have found both acute and chronic benefits of PA on cognitive and academic performance [11–13]. This research has primarily used cross-sectional study designs or been conducted in highly controlled laboratory-based or after-school settings focused on the quantitative aspects of PA such as intensity, frequency, and duration. This body of research has been summarized in several reviews concluding that most studies report either a positive relation of PA to academic performance, or no relationship between these constructs [2, 7, 11–15]. The positive relationship between PA and academic performance has also been demonstrated in school-based interventions lasting one-to-three years with a PA dose ranging from 60 to 120 min of extra moderate-to-vigorous PA (MVPA) per week [16–18]. In school settings, this type of PA has often been conducted outside of the academic sessions, such as before or after lessons, during PA breaks or at recess [7, 11, 13, 19].

An emerging line of research is rooted in embodied cognition theory, characterized in the education field as “embodied learning”. This research line focuses, for example, on gestures and bodily engagement closely related to and integrated into curricular learning tasks [20], and suggests that forming actions with the body can lead to the construction of enhanced mental representations that positively affect memory recall [21, 22]. This theory suggests that sensory-motor experiences can be linked to the cognitive processes involved in complex problem-solving and strategy use, and therefore, that PA can play a crucial role in the knowledge retention and retrieval [23–25]. This line of research has focused on the qualitative aspects of PA, such as cognitive and coordinative demands [26–28]. Some school-based studies have shown promising results on academic performance following interventions containing elements of embodied learning [4, 29, 30].

Therefore, the overall aim of the ACTIVE SCHOOL study is to investigate the effects of these two different types of PA interventions (MVPA and PA integrated in the learning tasks) on academic performance in Danish children aged of 9-10-years-old (3rd grade). Due to the challenge of implementing PA in schools, both interventions have been co-developed with school staff, as recommended [31]. The design phase is described in more details and published elsewhere [32]. Prior to commencing the randomized controlled trial (RCT), an 8-week feasibility study was set up to evaluate the implementation of the two interventions, and the feasibility of the proposed test battery for the RCT [33] Accordingly, the aim of this protocol is to summarize knowledge and experiences from the development phase, and to introduce the rationale and design of the cluster RCT-study.

## Methods

**Fig. 1:**
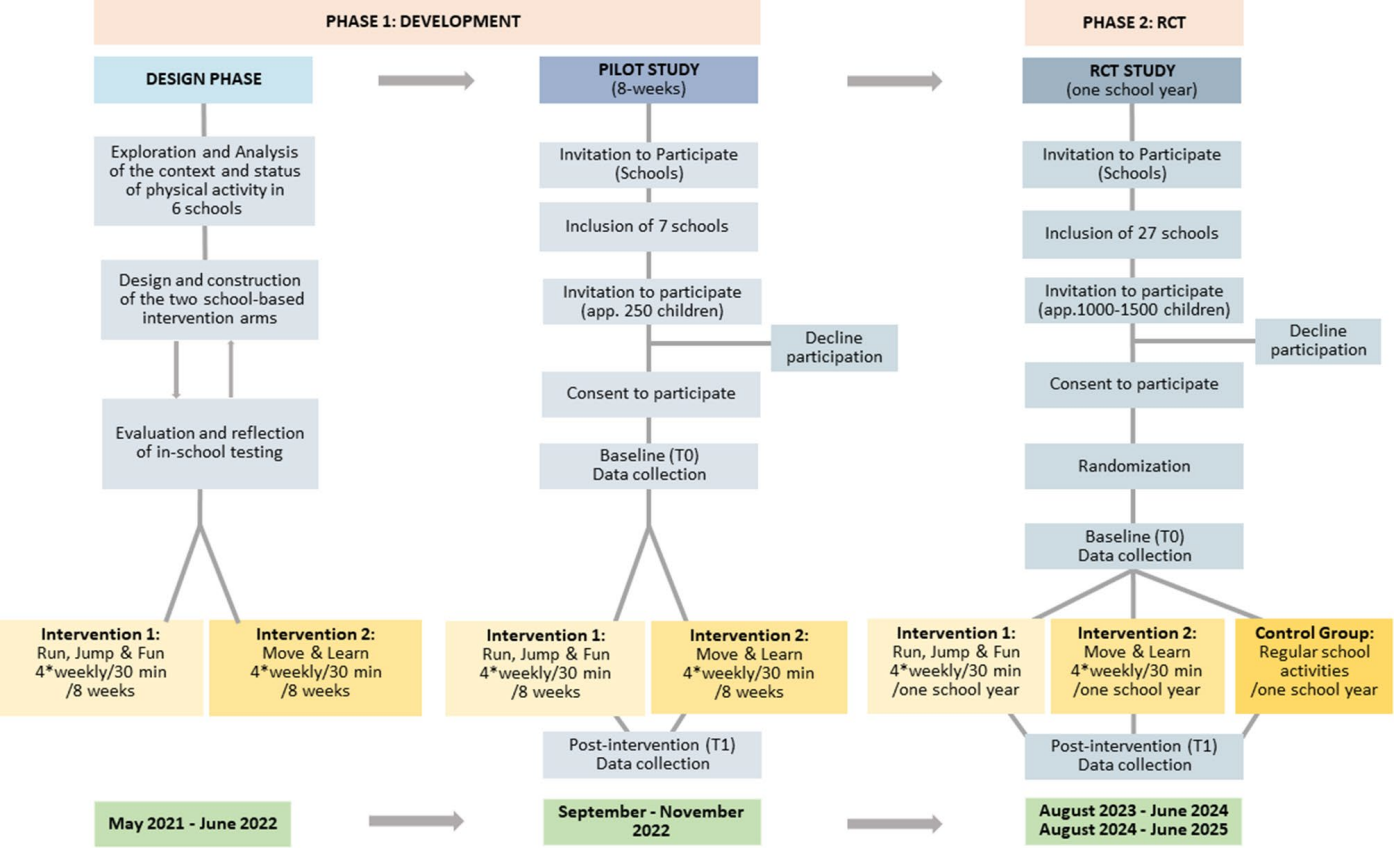
Phases and timeline in the ACTIVE SCHOOL study

The ACTIVE SCHOOL study consists of two phases: 1) The Development phase including the design phase and feasibility testing of the two PA interventions, and 2) the RCT investigating the effects of the interventions. The two phases of ACTIVE SCHOOL study are illustrated in Fig. 1.

### Design phase

The ACTIVE SCHOOL study’s design phase was conducted from May 2021 until June 2022 and is published elsewhere [32]. It was guided by McKenney and Reeves´ *Generic Model for conducting research in education* [34]. This model integrates theory, research, and practice into one model. The model comprises three core phases 1) Exploration and Analysis, 2) Construction and Design, and 3) Reflection and Evaluation. The two PA interventions ‘*Run, Jump & Fun*’ (RJF) and ‘*Move & Learn*’ (ML) were designed in collaboration between researchers and school staff, and the results were the intervention principles presented in Table 1. The intervention dose were defined as 30 min of PA delivered four times per week, with several implementation strategies to facilitate adoption and practice of the interventions [35].

**Table 1:** Principles of ‘*Run, Jump & Fun*’ and ‘*Move & Learn*’ interventions developed throughout the design phase and based on in-school testing, observations, and reflections in the researcher team

RJF principles	ML principles
• Connect a pedagogical aim to the RJF activity. • Pay attention to the target group and prepare activities in advance. • Instructions must be short and precise. • Modify the activities, so inactive breaks do not occur. • Be aware of the duration and intensity of activities. • Foster the possibility of autonomy in activities. • Discover opportunities for activities in alternative settings.	• Integrate curriculum-based PA in two Danish and two Math lessons per week. • The bodily engagement must underpin the curricular aim of the lesson. • The body can and must be a part of the immersion or the solution of an academic activity during the intervention time. • Organize group activities with a maximum of three members in each group. • Foster the possibility of autonomy in activities.

### Feasibility study

As recommended before performing an effect study, implementation outcomes (e.g., acceptability and feasibility) of the two interventions were assessed during an eight-week feasibility study conducted between September and November, 2022 [33, 36] (will be reported elsewhere – manuscript under preparation).

The aims of the feasibility study were to: 1) Investigate implementation outcomes of the RJF and ML interventions and, 2) Test the preliminary test-battery for the RCT-study.

The feasibility study resulted in the final definition of the two interventions, including principles, strategies, course designs, dose and frequencies (see *Intervention conditions* and *Training of school staff*). During the feasibility study the test-battery was tested at four schools to ensure that the final battery was applicable for the RCT, considering logistical factors such as resources, test days, duration, and feasibility. The feasibility of the test loop during the test day was examined, leading to necessary adjustments. Furthermore, optimization and standardization of test procedures were assessed, considering the duration of the testing process. Adjustments were made after the feasibility study, refining the test schedule, supplementing academic and executive function tests, and standardizing equipment for anthropometry in preparation for the RCT. Various approaches were explored for standardized academic outcomes, including evaluating mathematical tests for second and third-grade students to determine the appropriate academic level for the RCT. Teachers were found to be capable of conducting standardized academic tests.

During the feasibility study, challenges occurred in managing data across paper and various software platforms. To address this, we implemented REDCap (see *Methods, Outcome Measures*) to streamline data collection and handle extensive datasets.

### Randomized controlled trial (RCT)

The overall aim of the ACTIVE SCHOOL RCT study is to investigate the effects of two different types of in-school PA interventions on academic performance in children aged 9–10 years (3rd grade). The cluster RCT has three arms and includes two intervention groups; (1) RJF and (2) ML, and (3) control group (CG). The interventions are planned for one school year carried out in two rounds during the school years 2023/2024 and 2024/2025. The randomization will be stratified at the school level with an approximate 1:1:1 recruitment protocol (RJF:ML:CG). Approximately nine schools will be allocated to each arm (see *Randomization*). Due to the study design, blinding of instructors, teachers and participants will not be possible, which is common practice in school-based studies.

Schools from all regions of Denmark will be recruited by phone, e-mail, relevant websites, and digital/social media targeting school staff and school principals. If principals and school staff agree to participate in the study, an informal project meeting will be held, and comprehensive information will be provided.

#### Intervention conditions

##### Run, Jump & Fun intervention

RJF consists of four sessions of 30 min of MVPA each week for one school year. RJF must be conducted on all school days except the day timetabled with mandatory Physical Education (PE).

During two initial process meetings, the RJF intervention is established and tailored to fit the local school structure. ACTIVE SCHOOL project personnel, school principal and school staff take part in this establishing process, before the beginning of the intervention. RJF sessions can be placed in different time slots during the day, and should be delivered by either PE teachers, teachers from other subjects or the school’s pedagogical personnel (pedagogues). The intervention deliverers must engage in all RJF training and use the intervention materials (see below).

Besides the duration and intensity requirements, an additional aim of RJF activities is that they are fun, motivational, and inclusive for all children, regardless of academic skills, PA, fitness or motor skill level. To achieve this aim a motivational environment supportive of children’s´ needs will be created, as outlined in the self-determination theory [37]. The delivery of the intervention and practice with RJF will be guided by the seven principles developed in the design phase (see Table 1).

Examples of RJF activities include ball games in small teams, relay and obstacle run, martial art games or dance. RJF can be conducted in various settings depending on the individual school. Typically, RJF will take place in either the classroom or adjacent indoor spaces, or outdoor spaces (e.g. in the schoolyard, or in the gym hall).

##### Move & Learn intervention

ML consists of four sessions of 30 min of Physical Active learning (two math lessons and two Danish lessons) every week for one school year. ML will be conducted on all school days except the day timetabled with PE. All 3rd grade classes in the Danish school system have a minimum of four lessons (45 min each) of math and six lessons of Danish each week. ML lessons will be conducted in the classroom, adjacent spaces in the school building, outdoors in the school yard, or in the local environment. One initial process meeting with ACTIVE SCHOOL project personnel, school principal and school staff is held at each ML intervention school to establish and tailor the intervention to the local school structure.

ML is delivered by Danish and math teachers, who must engage in all ML training and use the intervention materials.

All ML lessons should focus on embodied learning, with a close connection between the academic learning task and bodily engagement. ML activities aim to be fun, motivating and foster mastery of the subject matter among the children. ML is guided by five principles developed in the design phase (see Table 1).

In ML activities could, for example, be group work dramatizing verbs with the whole body while reading a story out loud, using big grids in the school yard and let the children play an active figure in a coordinate system, or discovering ambience in a novel, by visiting and exploring places that sound, smell or feel like the place described by adjectives in the novel. Integration of the body in learning tasks can vary. It can be physical or motor-skill demanding activities, or it can be less vigorous activities, even standing up miming, using hand gestures or facial expressions.

##### Both interventions

To support implementation, school principals should agree to allocate 22 h to each teacher/pedagogue involved in delivering the interventions and 32 h for one teacher to coordinate with ACTIVE SCHOOL, e.g., set dates for courses and testing. Every teacher or pedagogue involved in the project will have these hours prioritized in their teaching load (their planned tasks during the year) to be able to participate in project training and meetings.

As a strategy to sustain the interventions for a whole year, each school organize school staff meetings about the intervention every second month to plan and share experiences about the intervention. Meeting agendas and guides will be provided by the ACTIVE SCHOOL project group. These meetings must be supported by school leaders who can attend or get minutes from the meeting.

#### Training of school staff

Teachers and pedagogues receive specific training for delivering either RJF or ML interventions. The training is conducted by educators from University College Copenhagen teacher education and pedagogue education. To ensure the sustainability of the interventions, a gradual introduction to the intervention principles has proven effective in previous studies [38–40]. Therefore, a gradual introduction will be applied in both interventions.

The RJF teacher training program includes a 6-hour introduction course held at a central location for all schools before the intervention starts, a 3-hour follow-up course held at each school after 2 months, and a 5-hour thematic follow-up course held at a central location for all schools after 6 months.

The ML teacher training program includes a 6-hour introduction course held at a central location for all schools before the intervention starts, a 3-hour follow-up course held at each school after 2 months, a 2-hour thematic follow-up course held in hybrid formats (on-site or online) at local schools after 4 months, and a 3-hour advanced course held at a central location for all schools after 7 months.

In both interventions, school staff are expected to organize internal team-meetings in the months without planned courses. These serve to maintain focus throughout the whole intervention period. A reflection paper will guide the internal staff-meetings in common reflections regarding progression and delivery of the interventions.

#### Control condition

The control schools will continue their normal practice but take part in all baseline- and post-testing of primary and secondary outcomes.

#### Recruitment of participants

Once schools agree to participate, all children and parents or legal guardians in participating classes will receive written information and consent forms from the research team. For a child to participate in the scientific measurements the parents/legal guardians must return a written informed consent prior to baseline measurement, but all children from intervention schools will participate in the intervention activities (See fig. 1: Phases and timeline in the ACTIVE SCHOOL study). Participation in the project is voluntary and free of charge, and it is possible to withdraw consent at any time without giving a reason.

There are no exclusion criteria for children in the participating classes. It is unlikely that adverse events and other unintended effects of the interventions will occur when using the described scientific methods. In the rare cases where incidental findings, like mis-thriving and anxiety may occur, the person responsible for the research will contact the school for further contact to parents/guardians and relevant authorities.

#### Randomization

An independent statistician employed at the Department of Mathematics, University of Copenhagen, facilitated the process of randomizing the participating schools into three groups. The statistician utilized the open-source software R and the sample function to generate a randomized list of numbers for the 27 schools. To ensure a balanced stratification, we sequentially assigned numerical labels to the schools based on their receipt of written consent for participation. The statistician remained unaware of the intervention content and the true identities of the schools. The schools enrolled in the study for the school year 2023/24 were informed of their assigned groups before the summer holiday starting in June 2023 and new schools for the school year 2024/25 will be informed before summer holiday 2024.

#### Sample size and power

Schools are the units of cluster-randomization. A conservative power estimation with an expected effect size of 0.42 for the main outcome (general math, based on results from [17]) and an intra class correlation of 0.15, resulted in a sample of ≥ 9 schools per intervention arm (27 in total), with a mean of 20 children per class and a minimum of one class per school (power 0.8, 1-tailed, α = 0.05). Mixed-effects models will be used to test the between-group difference (intervention vs. control) for the main outcome (general Math), controlling for baseline values and including school and class as random effects [41].

#### Outcome measures

To evaluate the effects of the interventions, children will be assessed before (T0) and after the intervention period (T1). Assessments will be carried out by research staff trained by senior research team members of ACTIVE SCHOOL and will be performed during school hours. Outcomes measured to assess potential effects of the two interventions (RJF and ML) include academic performance in math (primary outcome) and Danish, cognitive functions (working memory tasks), motivation for school, well-being, anthropometry, cardiorespiratory fitness, and physical activity level as secondary outcomes. Data entry will be performed onsite in REDCap using laptop computers and written on paper for random sample quality testing. REDCap is a secure web application for building and managing online surveys and databases [42]. Additionally, objective PA measures will be conducted at T0, T1 and at some schools also during the intervention. Implementation outcomes will be assessed on a regular basis (see below).

#### Academic performance tests

Standardized academic tests in Danish and math will be performed by school’s teachers, who will follow the test team’s written and oral instruction given in advance. The academic tests will be conducted early on the school day, before any school-based PA is initiated to avoid acute effects. The tests will be performed on computers in the classroom before the intervention start (T0) and in the end of the intervention period (T1). The children will be placed at distance to avoid copying.

Test results will be collected and administered by Hogrefe, a publisher of psychometric assessments and psychology books and journals. Data from tests will be securely transferred to ACTIVE SCHOOL research team and stored on a secure drive. The tests from Hogrefe are commonly used in educational practice in Denmark [43–46].

##### Standardized Test in Math

A standard, diagnostic, computerized test will be used to evaluate children’s math proficiency in relation to second (MAT2 assessed at T0) and third-grade (MAT3 at T1) proficiency [45]. Fifty problems from arithmetic (addition, subtraction, and multiplication), geometry, and probability will be included in the test. Children will be positioned in front of a computer, given the task of solving all 50 problems to the best of their abilities. The test is conducted during one school-week at several timeslots and managed to the manufactory description. The quantity of problems that are successfully solved will be the outcome indicator.

##### Standardized Reading Tests in Danish

Standardized Reading Comprehension Test: A valid and reliable Danish reading comprehension test (Sætningslæseprøve 2) will be used to evaluate the children’s reading abilities [44]. The test will be performed online and instructed by the Danish teacher. The test is appropriate for children in 3rd grade, and will be used both at baseline (T0) and at the end of the intervention (T1). The test includes 27 drawings of situations that are each accompanied by four statements. The child must check a true or false box next to each statement to indicate whether the text agree with the situation depicted in the drawing. The complexity and length of the sentences will increase gradually. Following the test protocol, eight minutes will be allotted for the child to complete as many statements as they can. The number of statements correctly evaluated will serve as the outcome.

##### Standardized Word Reading Test

The word reading test (Ordlæseprøven 2) assesses children’s word reading ability and will be administrated strictly according to the manufacturer’s description [46]. Within four minutes, children must read as many words as possible. The test is a multiple choice with four drawings for each printed word. Each child should read the word and choose the matching drawing. The test outcome is the number of correctly read words in four minutes.

#### Anthropometric and demographic variables

Participants’ height will be measured using a stadiometer (West Sussex, UK) with a precision of 0.5 cm. Body weight will be measured to one decimal (0.1 kg) using an electronic scale (Tanita WB-150 SMA, Tokyo, Japan) and the children will not be able to see their weight measure. Overweight and obesity will be determined based on age- and gender-specific body mass index (BMI) reference values [47]. Waist circumference will be measured to the nearest 0.5 cm above the navel, with a minimum of two measurements taken. If the measurements differ by more than 2.0 cm, a third measurement will be performed and the average of the two closest measurements will be used. Participants will self-report their puberty stage using a questionnaire, which consists of five categories of secondary sex characteristics defined by Tanner [48], using standardized color images as suggested by Carel and Leger [49]. Information on pubic hairs development in boys, and menstruation (yes/no), breast and pubic hair development in girls will be collected.

#### Cognitive tests

The cognitive test battery includes visuo-spatial working memory and a 1-back working memory task.

##### Visuo-Spatial Working Memory Task

Visuo-Spatial Working Memory (VSWM) task is a self-ordered search task, which assesses an individual’s ability to store and manipulate information about the location of objects on a computer screen [50]. First, the test displays three filled circles on a computer screen (level 3). The participant must find a red dot behind one of the circles by touching each of the circles one at the time. The objective is to touch as few times as possible at each level. The red dot can only be positioned behind one circle and must be dragged to a column on the right side of the screen, when found. The red dot cannot be placed behind the circle more than one time at a given level. When a given level is finished, the participant continues to the next level where there are additional number of circles. The number of circles increases until there are ten circles on the screen. The placement of the circles is randomly distributed from trial to trial. The test measures time to complete each level, the number of errors made and the number of times the participant changes search strategy during the trials. Errors are defined as touching circles where the red dot cannot be (i.e., either previously found or already touched). The outcome measure will be a score based on time, error and strategy during the last three levels.

##### 1-back working memory task

Updating of working memory is assessed using a 1-back task. This 1-back task is constructed of representations of symbols (e.g., car, cloud, key, eye and bicycle); one symbol is presented at the time. The child must compare the symbol seen on the screen with the symbol previously presented. Children are instructed to press “yes” (a green key) on the keyboard if these two symbols are identical and “no” (a red key) if they are dissimilar, as fast, and as accurate as possible. The test consists of 20 practice trials followed by one test block of 20 trials (both 30% “yes” trials). Symbols are presented for 500 ms followed by a 3000 ms blank screen. The response window and inter-stimuli interval is 3500 ms each. The results from the test constitute number of correct and wrong “yes” and “no” answers, mean reaction time for correct “yes” and “no” answers, and number of non-responses in the test block. Normative data on the n-back task for children and young adolescents have previously been reported [51].

#### Physical Tests

##### Andersen Test

Cardiorespiratory fitness will be measured using the Andersen-test, a 10-minute intermittent running test (15 s running and 15 s pausing), running back and forth on a 20 m track [52]. Total distance covered will be used to represent cardiorespiratory fitness. Verbal encouragement is given to encourage maximal performance. If an obvious submaximal performance by a child is seen, the results will be discarded. This test has been proven valid and reliable compared to direct measures of maximum oxygen uptake in a similar cohort [53]. The Andersen-test has demonstrated reliability and validity for the target age group [54].

##### Accelerometry

PA will be assessed using accelerometer measurements (Axivity, Newcastle, UK) for one week before the intervention starts (T0), once during the intervention at randomly selected schools (but not the full sample), and one week for all schools towards the end of the intervention (T1). Children will wear the accelerometer for seven consecutive days taped on their right thigh. Data for a full day will be analyzed with minimum wear-time requirements of 8 h/day. ACTi 4 software (Version 14.09, ACTi Corp., Copenhagen, Denmark) will be used to identify different types of PA such as sitting, standing, walking, fast walking, running, cycling, sit-to-stand movements, and steps taken. The amount of time spent on each activity type each day will be calculated and time spent in different PA intensity levels according to the recommendations will be reported [55].

#### Wellbeing

Children’s wellbeing and quality of life will be assessed using the Kidsscreen-27 questionnaire. The Kidsscreen-27 is a valid and reliable tool for measuring health-related quality of life and wellbeing in children and adolescents. The questionnaire has been used in various research projects to gain insights into the subjective experiences and perceptions of young people regarding their quality of life and well-being [56]. Kidscreen-27 assesses the following 5 domains of health related to quality of life: physical well-being (5 items), psychological well-being (7 items), relation to parents and autonomy (7 items), social support and peers (4 items), and the school environment (4 items). The original questionnaire consists of 27 items and is structured in a series of questions answered on a five-point Likert scale, ranging from “never” to “always”. As this intervention is school-based only, the parent and the autonomy domain are not included in this study. The questionnaire will be administered in a paper version and every item will be read aloud for the children to overcome any reading difficulties.

#### Motivation

Children’s motivation for school will be assessed with the Self-Regulation Questionnaire-Academic (SRQ-A) [57]. SRQ-A is a domain specific, self-reported questionnaire developed and validated for children in primary and lower secondary school [37]. The SRQ-A originally measures four types of motivation (or behavior regulation) for engaging in schoolwork and learning activities identified in the Self Determination Theory; intrinsic motivation, identified regulation, introjected regulation and external regulation. However, in this study only the two autonomous types of motivation; intrinsic motivation and identified regulation, will be measured to shorten the questionnaire to10 questions. The questionnaire will be administrated electronically in REDCap. A trained person will aid the children with reading each item while they complete the questionnaire on a computer.

#### Implementation process evaluation

To evaluate the implementation of the interventions, the Reach, Effectiveness, Adoption, Implementation, and Maintenance (RE-AIM) framework will be used as a guide [58]. The RE-AIM framework is widely used to plan, guide and evaluate intervention studies and expand the knowledge of “how and why”. Definitions of the RE-AIM implementation outcomes adapted to ACTIVE SHOOL are displayed in Table 2. The outcomes will be assessed both quantitative and qualitatively and will be collected on multiple levels (child, teacher, school management).

**Table 2:** Dimension definitions of RE-AIM in ACTIVE SCHOOL process evaluation

Evaluation dimensions definitions of RE-AIM	
**Dimension**	**Definition**
Reach	Refers to recruitment strategy and procedure for this. Refers to the proportion and representativeness of included schools and children.
Effectiveness	Refers to the effect of the interventions on academic performance, cognitive performance, motivation and well-being in intervention group children compared to the control group, who do not receive the intervention.
Adoption	Refers to the principal’s decision to accept the intervention as a part of a school year, and staff decision to adopt the intervention or not. This is reported as the acceptability, appropriateness rated by the staff and their delivery of any part of the intervention.
Implementation	Refers to the staff adherence to the target minutes of the intervention and the fidelity to intervention principles delivered as intended. Feasibility and adaptability additionally refer to implementation quality.
Maintenance	Refers to the extent to which intervention implementation was maintained throughout the school year (short-term maintenance). Additionally, indicators of sustainability are identified.

##### Reach

In the present study, the reach dimension reports how the recruitment of schools is executed, as well as the size and representativeness of the included school. Also, the strategy for recruiting children is reported. Reach also covers demographic description of parents/guardians of the participating children and the teachers, where the proportion of consents returned will be a measure of willingness and commitment to the project.

##### Effectiveness

Effectiveness is to which extend the interventions affect academic, cognitive, physical and motivation outcomes, as described in *Outcome Measures*.

##### Adoption

Adoption is a measure of the acceptability and understanding of the interventions experienced by the school staff assessed both quantitatively and qualitatively.

Teachers and pedagogues will complete the survey Usage Rating Profile-Intervention (URP-I) before the interventions begin, midway and at the end of the intervention period [59]. URP-I is a validated, self-reported questionnaire containing 29 items covering six factors: acceptability, understanding, feasibility, system climate, and system support. Each item is rated on a scale from 1 (Strongly disagree) to 6 (Strongly agree). A summed score will be calculated for each of the factors. The original URP-I is translated into Danish following a protocol of a four-step-process inspired by WHO guidelines [60].

Additionally, adoption will be evaluated by interviews with school staff involved in the study. Teachers, pedagogues, and school principals will be invited to focus group interviews. The purpose of the interviews is to gain deeper understanding of how the interventions were adopted into practice and which adaptations were made. Interviews will be conducted in the last months of the intervention. The interviews will follow a semi-structured guide, be transcribed verbatim and undergo reflexive thematic analysis [61].

##### Implementation

The implementation evaluation will give comprehensive knowledge of how the interventions are implemented in the schools and how they are adapted to the specific schools’ context. Implementation fidelity will be evaluated in a self-reported SMS survey sent out every week throughout the study period. The teachers/pedagogues will receive a SMS with a link to a short survey, which can be answered from their mobile phones. These surveys will be distributed, and data will be collected and stored using REDCap. The survey questions will assess adherence in all three groups (RJF, ML and CG), in terms of the total minutes of PA delivered. In the two intervention groups fidelity will be measured, in terms of how closely the intervention principles were followed over the previous week. The feasibility of the implemented strategies will also be measured with the URP-I questionnaire and within the aforementioned focus group interviews. Furthermore, observations will be conducted in the schools [62].

##### Maintenance

Maintenance will be measured six months after the beginning of an intervention and onwards [63]. Answers on delivery of minutes of PA from SMS survey will be the outcome. Furthermore, group interviews will cover intentions to maintain the interventions after the end of the intervention period.

#### Plan for data management, analysis, and statistics

Descriptive statistics across groups will be summarized and presented. Flow diagram of participants will be described and presented according to the Consolidated Standards of Reporting Trials (CONSORT) 2010 for randomized controlled trials [64]. Differences at baseline between participants included and excluded in the main analyses will be presented (e.g., drop-out analyses) and data will be analyzed for between-group differences (interventions vs. control) at baseline for all outcomes. The effects of the intervention on both primary and secondary outcomes will be tested in intention-to-treat analysis using multilevel mixed models with group (interventions/control) as fixed effect and adjusted for relevant confounders not evenly distributed between groups at baseline. School and class will be included as random effects in the model to account for the cluster structure of the data. Furthermore, per-protocol analysis will be performed including only children from classes compliant with the intervention principles (based on SMS-survey answers from teachers/pedagogues). The statistical analysis will be performed using R (R Core Team, 2022) and in the IMB Statistical Package for the Social Sciences (SPSS) version 24.

#### Data security

All participating children in the study will be anonymized by an identification number (ID number), and no identifying information will be stored. A key coupling the ID number with personal information will be stored separately to secure confidentiality. All data will be entered, secured and stored in REDCap. The data management plan is available on request. Only researchers participating in the ACTIVE SCHOOL project will have access to the final dataset. Other researchers can apply for the use of completely anonymized data after contractual agreements have been made. All rules from the Danish Data Protection Agency and General Data Protection Regulation (GDPR) will be followed. Results from the project will be communicated to the participating families and schools, the press, and will furthermore be published in non-scientific journals, as well as in peer-reviewed, scientific journals. Authorship will follow the Vancouver Recommendations.

#### Organizational structure and responsibilities

The research team behind the ACTIVE SCHOOL project is a multidisciplinary group of experts in PA, school context, cognition, learning, motivation and childhood health. This team is responsible and handles all aspects of the study management including recruitment, training of intervention deliverers and test personnel, data collection and handling, final analysis and reporting of results. Since the ACTIVE SCHOOL project was initiated, an international multidisciplinary advisory board has been assigned. The board has ensured scientific quality in all aspects of the project.

## Discussion

The school setting is well suited for large-scale PA initiatives. In Denmark, most children and adolescents attend school, therefore, school-based PA interventions have the potential to reach almost all children including overweight, physically inactive, and unfit children, who are difficult to target by other means. Enhanced PA might have a positive impact not only on health and wellbeing [3, 19], but also on cognitive skills and academic performance [7, 8, 11–13, 26].

Despite a growing body of evidence connecting PA in schools with enhanced academic performance, it remains unclear which specific forms of PA in the school are the most feasible and effective for the benefit of children’s academic performance. Following the development and feasibility study, the ACTIVE SCHOOL RCT study will assess the feasibility and effects of the two interventions, RJF and ML, focusing on two different means to enhance PA in schools. Only few school-based studies have compared different approaches to implement PA during the school day. The Norwegian ‘School in Motion Study’ investigated effects on academic performance of two different interventions on Norwegian 14-year-olds [6]. One intervention focused on increasing the students’ PA level (called *Physically Active Learning*) and another group focused on promoting friendship and collaboration between students (called *Don’t worry – Be Happy*). Both intervention groups improved academic performance in math compared to the control group, demonstrating that different PA interventions in schools can be viable models to increase academic performance [6]. Like the ‘School in Motion Study’, ACTIVE SCHOOL aims to assess the feasibility and test the effects of two different approaches to enhance PA in schools.

### Strength and Limitations

The ACTIVE SCHOOL study has several strengths. First, the two interventions were developed in collaboration with school staff, which should increase the chances of acceptability and feasibility [33, 65]. Second, the feasibility-study enabled optimization of the protocol and design for successful implementation of the interventions in the school setting [33, 35]. Third, to facilitate successful adoption, fidelity and long-term sustainability of the interventions, the study provides teachers and pedagogues with support for establishing the interventions, training courses, teaching materials and follow-up meetings throughout the one-year implementation period. Collectively, these strategies should enhance the feasibility and effectiveness of the interventions in the school context [35].

An additional strength is the application of the SPIRIT 33-item checklist and thereby following the recommendations for reporting [66] (see Supplementary Material 1). Finally, the process evaluation guided by the well-documented and comprehensive RE-AIM framework is considered a strength of the study ensuring assessments of relevant implementation outcomes, advancing the knowledge gained in the study [58, 67, 68].

There are several limitations. Despite using a well-described and commonly used test in Denmark for the main outcome (academic performance in math), the fact that the teachers administer the test in their classroom may be considered a limitation and may potentially cause data-collection bias [46]. Also, the choice to not include other tests of academic and cognitive performance may be a limitation. However, this decision was based on real-world time constraints that exist when conducting research in the classroom, as caution is needed so as not to burden schools and teachers.

An additional limitation is that the school setting is a difficult environment to conduct research, as schools are dynamic, complex, and unpredictable. Every school is unique, and schools vary in size, type, student composition, teacher resources, budget (amount and prioritizing), urban/rural uptake area, physical environment, school culture, etc. Therefore, standardization of a school PA interventions is not easily achievable. To account for these differences, interventions for each participating school were adapted and tailored to fit each school’s unique context to optimize implementation [69–72].

Schools voluntarily indicated their willingness to participate in the study, in many cases because they have a profound interest in PA. Their decision to participate could be motivated by their desire to introducing PA into their curriculum or to strengthen an existing culture of PA. In the latter case, the implications could be a reduced probability of finding an effect of the intervention.

The ACTIVE SCHOOL study will contribute to the current knowledge regarding the feasibility and effects on academic performance of two different approaches to enhance PA in schools (Physical activity with a focus on having moderate-to-vigorous intensity vs. movement activities aimed at embodied learning of math and Danish) when implemented in real world scholastic settings. By doing so, the study has the potential to contribute to future research, as well as educational practice on using PA in schools to support education and learning.

### Electronic supplementary material

Below is the link to the electronic supplementary material.

**Supplementary Material 1:** SPIRIT 2013 Checklist: Recommended items to address in a clinical trial protocol and related documents

**Supplementary Material 2:** 1. Written information to parents/guardians, 2. Consent form for parents, 3. Written information to teachers, 4. Consent form for teachers and, 5. ACTIVE SCHOOL SPIRIT diagram displaying study recruitment, intervention and measures schedule

## Data Availability

Not applicable.

## Abbreviations

PA: Physical activity
MVPA: Moderate to vigorous Physical activity
RJF: ‘Run, Jump & Fun’ intervention
ML: ‘Move & Learn’ intervention
RCT: Randomized Controlled Trial
CG: Control group
PE: Physical Education
BMI: Body mass index
VSWM: Visiou-Spatial Working Memory
SMS: Short Message Service
SEM: structural equation models

## References

[1] Moore PJ (2019). Academic achievement and social and emotional learning. Educational Psychol.

[2] Donnelly JE, Ed D, Co-chair F, Hillman CH, Co-chair PD, Ph D et al. Physical activity, fitness, cognitive function, and academic achievement in children: a systematic review. 48, Medicine and Science in Sports and Exercise. 2016. 1197–222 p. 10.1249/MSS.0000000000000901. 10.1249/MSS.0000000000000901 PMC4874515 27182986

[3] Bangsbo J, Krustrup P, Duda J, Hillman C, Andersen LB, Weiss M et al. The Copenhagen Consensus Conference. 2016: children, youth, and physical activity in schools and during leisure time. Br J Sports Med 2016;50(19):1177–8. Available from: 10.1136/bjsports-2016-096325. 10.1136/bjsports-2016-096325 PMC5036221 27354718

[4] Have M, Nielsen JH, Ernst MT, Gejl AK, Fredens K, Grøntved A et al. Horn L, editor. PLoS One. Classroom-based physical activity improves children’s math achievement – A randomized controlled trial. Van 2018;13(12):e0208787. 10.1371/journal.pone.0208787. 10.1371/journal.pone.0208787 PMC6296522 30557397

[5] Mullender-Wijnsma MJ, Hartman E, de Greeff JW, Bosker RJ, Doolaard S, Visscher C (2015). Moderate-to-vigorous physically active academic lessons and academic engagement in children with and without a social disadvantage: a within subject experimental design. BMC Public Health.

[6] Solberg R, Steene-Johannessen J, Anderssen SA, Ekelund U, Säfvenbom R, Haugen T (2021). Effects of a school-based physical activity intervention on academic performance in 14-year old adolescents: a cluster randomized controlled trial – the School in Motion study. BMC Public Health.

[7] Singh AS, Saliasi E, van den Berg V, Uijtdewilligen L, de Groot RHM, Jolles J (2019). Effects of physical activity interventions on cognitive and academic performance in children and adolescents: a novel combination of a systematic review and recommendations from an expert panel. Br J Sports Med.

[8] Norris E, van Steen T, Direito A, Stamatakis E. Physically active lessons in schools and their impact on physical activity, educational, health and cognition outcomes: a systematic review and meta-analysis. Br J Sports Med. 2020;54(14):826 LP – 838. 10.1136/bjsports-2018-100502. 10.1136/bjsports-2018-100502 31619381

[9] Gomez-Pinilla F, Hillman C. The influence of Exercise on cognitive abilities. Comprehensive Physiology. Wiley; 2013. pp. 403–28. 10.1002/cphy.c110063. 10.1002/cphy.c110063 PMC3951958 23720292

[10] Thomas A, Dennis A, Bandettini P, Johansen-Berg H. The effects of aerobic activity on Brain structure. Front Psychol. 2012;3. 10.3389/fpsyg.2012.00086. 10.3389/fpsyg.2012.00086 PMC3311131 22470361

[11] Marques A, Santos DA, Hillman CH, Sardinha LB (2018). How does academic achievement relate to cardiorespiratory fitness, self-reported physical activity and objectively reported physical activity: a systematic review in children and adolescents aged 6–18 years. Br J Sports Med.

[12] Fedewa AL, Ahn S (2011). The effects of physical activity and physical fitness on children’s achievement and cognitive outcomes: a Meta-analysis. Res Q Exerc Sport.

[13] Watson A, Timperio A, Brown H, Best K, Hesketh KD (2017). Effect of classroom-based physical activity interventions on academic and physical activity outcomes: a systematic review and meta-analysis. Int J Behav Nutr Phys Act.

[14] Álvarez-Bueno C, Pesce C, Cavero-Redondo I, Sánchez-López M, Garrido-Miguel M, Martínez-Vizcaíno V. Academic achievement and physical activity: a Meta-analysis. Pediatrics. 2017;140(6). 10.1542/peds.2017-1498. 10.1542/peds.2017-1498 29175972

[15] Macdonald K, Milne N, Orr R, Pope R. Relationships between Motor Proficiency and Academic Performance in Mathematics and Reading in School-aged children and adolescents: a systematic review. Int J Environ Res Public Health. 2018;15(8). 10.3390/ijerph15081603. 10.3390/ijerph15081603 PMC6121293 30060590

[16] Solberg R, Steene-Johannessen J, Ekelund U, Lerum Ø, Åvitsland A, Haugen T (2020). Effect of a School-based physical activity intervention on academic performance in Norwegian adolescents: the School in Motion Study - A Cluster Randomized Controlled Trial. Med Sci Sport Exerc.

[17] Mullender-Wijnsma MJ, Hartman E, de Greeff JW, Doolaard S, Bosker RJ, Visscher C (2016). Physically active Math and Language lessons improve academic achievement: a Cluster Randomized Controlled Trial. Pediatrics.

[18] Donnelly JE, Hillman CH, Greene JL, Hansen DM, Gibson CA, Sullivan DK (2017). Physical activity and academic achievement across the curriculum: results from a 3-year cluster-randomized trial. Prev Med (Baltim).

[19] Donnelly JE, Hillman CH, Castelli D, Etnier JL, Lee S, Tomporowski P (2016). Physical activity, fitness, cognitive function, and academic achievement in children: a systematic review. Med Sci Sports Exerc.

[20] Skulmowski A, Rey GD (2018). Embodied learning: introducing a taxonomy based on bodily engagement and task integration. Cogn Res Princ Implic 2018 31.

[21] Engelkamp J, Zimmer HD (1989). Memory for action events: a new field of research. Psychol Res.

[22] Barsalou LW. Perceptions of perceptual symbols. Vol. 22, behavioral and Brain sciences. Cambridge University Press; 1999. pp. 637–60.

[23] Barsalou LW (2008). Grounded Cognition. Annu Rev Psychol.

[24] Glenberg AM (2010). Embodiment as a unifying perspective for psychology. Wiley Interdisciplinary Reviews: Cogn Sci.

[25] Goldin-Meadow S, Nusbaum H, Kelly SD, Wagner S (2001). Explaining Math: gesturing Lightens the load. Psychol Sci.

[26] Norris E, Shelton N, Dunsmuir S, Duke-Williams O, Stamatakis E (2015). Physically active lessons as physical activity and educational interventions: a systematic review of methods and results. Prev Med (Baltim).

[27] Pesce C (2012). Shifting the Focus from Quantitative to qualitative Exercise characteristics in Exercise and Cognition Research. J Sport Exerc Psycho.

[28] Tomporowski PD, Pesce C (2019). Exercise, sports, and performance arts benefit cognition via a common process. Psychol Bull.

[29] Schmidt M, Benzing V, Wallman-Jones A, Mavilidi M-F, Lubans DR, Paas F (2019). Embodied learning in the classroom: effects on primary school children’s attention and foreign language vocabulary learning. Psychol Sport Exerc.

[30] Mavilidi MF, Lubans DR, Miller A, Eather N, Morgan PJ, Lonsdale C (2020). Impact of the thinking while moving in English intervention on primary school children’s academic outcomes and physical activity: a cluster randomised controlled trial. Int J Educ Res.

[31] Daly-Smith A, Quarmby T, Archbold VSJ, Corrigan N, Wilson D, Resaland GK (2020). Using a multi-stakeholder experience-based design process to co-develop the creating active schools Framework. Int J Behav Nutr Phys Act.

[32] Sohl Jeppesen L, Bugge A, Smedegaard S, Wienecke J, Sandfeld Melcher J. Developing ACTIVE SCHOOL – the design process of two School-based physical activity interventions. Transl J Am Coll Sport Med. 2024 (In press).

[33] Skivington K, Matthews L, Simpson SA, Craig P, Baird J, Blazeby JM et al. A new framework for developing and evaluating complex interventions: update of Medical Research Council guidance. BMJ. 2021;n2061. 10.1136/bmj.n2061. 10.1136/bmj.n2061 PMC8482308 34593508

[34] McKenney S, Reeves TC. Conducting Educational Design Research. Conducting Educational Design Research; 2018.

[35] Kirchner JAE, Smith JL, Powell BJ, Waltz TJ, Proctor EK (2020). Getting a clinical innovation into practice: an introduction to implementation strategies. Psychiatry Res.

[36] Pearson N, Naylor P-J, Ashe MC, Fernandez M, Yoong SL, Luke Wolfenden (2020). Guidance for conducting feasibility and pilot studies for implementation trials. Pilot Feasibility Stud.

[37] Ryan RM, Deci EL (2000). Self-determination theory and the facilitation of intrinsic motivation, social development, and well-being. Am Psychol.

[38] Lerum Ø, Bartholomew J, McKay H, Resaland G, Tjomsland H, Anderssen S (2019). Active smarter teachers: Primary School teachers’ perceptions and maintenance of a School-based physical activity intervention. Transl J Am Coll Sport Med.

[39] Resaland G, Moe V, Aadland E, Steene-Johannessen J, Glosvik Ø, Andersen H, et al. Active smarter kids (ASK): Rationale and design of a cluster-randomized controlled trial investigating the effects of daily physical activity on children’s academic performance and risk factors for non-communicable diseases. BMC Public Health. 2015;15(1). 10.1186/s12889-015-2049-y. 10.1186/s12889-015-2049-y PMC4517398 26215478

[40] Skage I, Ertesvåg SK, Roland P, Dyrstad SM (2020). Implementation of physically active lessons: a 2-year follow-up. Eval Program Plann.

[41] Athey S, Imbens GW. The Econometrics of Randomized Experiments. In: Banerjee AV, Duflo EBT-H of EFE, editors. Handbook of Field Experiments. North-Holland; 2017. p. 73–140. 10.1016/bs.hefe.2016.10.003.

[42] REDCap. Available from https://www.project-redcap.org/.

[43] Hogrefe. Available from: https://www.hogrefe.com/uk/.

[44] Lene Møller og Holger Juul. Hogrefe Psykologisk Forlag. Sætningslæseprøve 1 og 2. 2019. p. 1.

[45] Hansen K. MG/FG 3: Matematik grundlæggende færdigheder: Vejledning. Hogrefe Psykol Forl; 2012.

[46] Juul H, Møller L. Vejledning til Ordlæseprøve 1–2. Hogrefe Psykologisk Forlag; 2010.

[47] Cole TJ, Bellizzi MC, Flegal KM, Dietz WH (2000). Establishing a standard definition for child overweight and obesity worldwide: International survey. Br Med J.

[48] Tanner JM (1981). Growth and maturation during adolescence. Nutr Rev.

[49] Carel J-C, Léger J, Precocious Puberty (2008). N Engl J Med].

[50] Luciana M, Nelson CA (2002). Assessment of neuropsychological function through Use of the Cambridge Neuropsychological Testing Automated Battery: performance in 4- to 12-Year-old children. Dev Neuropsychol.

[51] Pelegrina S, Lechuga MT, García-Madruga JA, Elosúa MR, Macizo P, Carreiras M, et al. Normative data on the n-back task for children and young adolescents. Front Psychol. 2015;6. 10.3389/fpsyg.2015.01544/abstract. 10.3389/fpsyg.2015.01544 PMC4597481 26500594

[52] Andersen LB, Andersen TE, Andersen E, Anderssen SA. An intermittent running test to estimate maximal oxygen uptake: The Andersen test. J Sports Med Phys Fitness. 2008;48(4):434–7. Available from: https://pubmed.ncbi.nlm.nih.gov/18997644/. 18997644

[53] Ahler T, Bendiksen M, Krustrup P, Wedderkopp N, George KP (2012). Aerobic fitness testing in 6- to 9-year-old children: reliability and validity of a modified Yo-Yo IR1 test and the Andersen test. Eur J Appl Physiol.

[54] Aadland E, Terum T, Mamen A, Andersen LB, Resaland GK. The Andersen Aerobic Fitness Test: Reliability and Validity in 10-Year-Old Children. Johannsen NM, editor. PLoS One. 2014 ;9(10):e110492. 10.1371/journal.pone.0110492. 10.1371/journal.pone.0110492 PMC4201545 25330388

[55] Skotte J, Korshøj M, Kristiansen J, Hanisch C, Holtermann A (2014). Detection of physical activity types using Triaxial Accelerometers. J Phys Act Heal.

[56] Ravens-Sieberer U, Auquier P, Erhart M, Gosch A, Rajmil L, Bruil J (2007). The KIDSCREEN-27 quality of life measure for children and adolescents: psychometric results from a cross-cultural survey in 13 European countries. Qual Life Res.

[57] Ryan RM, Connell JP (1989). Perceived locus of causality and internalization: examining reasons for acting in two domains. J Pers Soc Psychol.

[58] Glasgow RE, Harden SM, Gaglio B, Rabin B, Smith ML, Porter GC, et al. RE-AIM planning and evaluation Framework: adapting to New Science and Practice with a 20-Year review. Front Public Heal. 2019;7. 10.3389/fpubh.2019.00064/full. 10.3389/fpubh.2019.00064 PMC6450067 30984733

[59] Briesch AM, Chafouleas SM, Neugebauer SR, Riley-Tillman TC (2013). Assessing influences on intervention implementation: revision of the usage Rating Profile-intervention. J Sch Psychol.

[60] Elsborg P, Melby PS, Kurtzhals M, Tremblay MS, Nielsen G, Bentsen P (2021). Translation and validation of the Canadian assessment of physical literacy-2 in a Danish sample. BMC Public Health.

[61] Braun V, Clarke V. Thematic Analysis. 1st ed. SAGE Publications Ltd; 2021. 376 p. Available from: https://study.sagepub.com/thematicanalysis.

[62] Lashley MC. Observational research methods. In: The SAGE Encyclopedia of Communication Research Methods. Thousand Oaks: SAGE Publications, Inc; 2017. p. 1116–8. Available from: https://sk.sagepub.com/reference/the-sage-encyclopedia-of-communication-research-methods.

[63] King DK, Shoup JA, Raebel MA, Anderson CB, Wagner NM, Ritzwoller DP, et al. Planning for implementation success using RE-AIM and CFIR frameworks: a qualitative study. Front Public Heal. 2020;8(0). 10.3389/fpubh.2020.00059. 10.3389/fpubh.2020.00059 PMC7063029 32195217

[64] Moher D, Hopewell S, Schulz KF, Montori V, Gotzsche PC, Devereaux PJ (2010). CONSORT 2010 explanation and elaboration: updated guidelines for reporting parallel group randomised trials. BMJ.

[65] Daly-Smith A, Morris JL, Norris E, Williams TL, Archbold V, Kallio J (2021). Behaviours that prompt primary school teachers to adopt and implement physically active learning: a meta synthesis of qualitative evidence. Int J Behav Nutr Phys Act.

[66] Chan A-W, Tetzlaff JM, Altman DG, Laupacis A, Gøtzsche PC, Krleža-Jerić K (2013). SPIRIT 2013 Statement: defining standard protocol items for clinical trials. Ann Intern Med.

[67] Nilsen P (2015). Making sense of implementation theories, models and frameworks. Implement Sci.

[68] Proctor E, Silmere H, Raghavan R, Hovmand P, Aarons G, Bunger A (2011). Outcomes for implementation research: conceptual distinctions, Measurement challenges, and Research Agenda. Adm Policy Ment Heal Ment Heal Serv Res.

[69] Brown KM, Elliott SJ (2015). It’s not as Easy as just saying 20 minutes a day’: exploring teacher and principal experiences implementing a Provincial Physical Activity Policy. Univers J Public Heal.

[70] Durlak JA, DuPre EP (2008). Implementation matters: a review of Research on the influence of implementation on Program outcomes and the factors affecting implementation. Am J Community Psychol.

[71] Koch S, Troelsen J, Cassar S, Pawlowski CS (2020). Barriers to implementation of physical activity in Danish public schools. J Teach Phys Educ.

[72] Nielsen JV, Bredahl TVG, Bugge A, Klakk H, Skovgaard T (2019). Implementation of a successful long-term school based physical education intervention: exploring provider and programme characteristics. Eval Program Plann.

