# Supplementary material for: Study protocol for the ACTIVE SCHOOL study investigating two different strategies of physical activity to improve academic performance in Schoolchildren
Source: BMC Pediatr. 2024 Mar 9;24:174. doi: 10.1186/s12887-024-04647-9 (PMC10924402; doi:10.1186/s12887-024-04647-9)
Supplement: Supplementary file 2 — Supplementary Material 2: 1. Written information to parents/guardians, 2. Consent form for parents, 3. Written information to teachers, 4. Consent form for teachers and, 5. ACTIVE SCHOOL SPIRIT diagram displaying study recruitment, intervention and measures schedule [file 12887_2024_4647_MOESM2_ESM.docx]

# **Appendix**

The appendix contains the following files:

1. Written information to parents/guardians
2. Consent form for parents
3. Written information to teachers
4. Consent form for teachers
5. ACTIVE SCHOOL SPIRIT diagram displaying study recruitment, intervention and measures schedule.
6. **Written information to parents/guardians**

**Participant and parent information**

**Title: *“ACTIVE SCHOOL*** *for a whole school year****”***

Dear parents of 3^rd^ grade students,

We are writing to ask if your child would like to participate in a scientific research project that aims to investigate how schoolchildren can be more active during the school day, with possible positive effects on learning outcomes, motivation, well-being, and health.

**The purpose** of the project is primarily to investigate how various types of physical activity during the school day can be arranged most effectively to motivate children and teachers and how it affects learning.

Plan for the project:

- Your child will participate in a program for one school year. Your child will either have classes with 1) movement incorporated into Danish twice a week and movement incorporated into math twice a week (Move & Learn), or 2) physical activities four times a week (Run, Jump & Fun) or 3) without any changes to normal classes (control class). All children in the participating classes will be involved in these activities.

Plan for the research part of the study:

- The research part of the study – the part of the project we are asking for your child's informed consent to participate in - involves data collection before, during, and after the program. Information will be gathered through questionnaires about motivation for movement and physical activity (what makes movement fun), as well as motivation for school in general. Your child will also complete a well-being questionnaire. Additionally, your child will participate in a fitness test in the gym/hall with classmates, and will undergo tests in Danish and math, as well as assessment of their cognitive function. Participating children will be introduced to and guided through all procedures before the actual data collection to ensure they feel comfortable and familiar with the exercises.

The research part of the project is organized by associate professor (docent) Anna Bugge, project manager Linn Damsgaard and associate professor Malene Norup Stolpe from University College Copenhagen, Department of Midwifery, Physiotherapy, Occupational Therapy and Psychomotor Therapy, together with associate professor Jacob Wienecke and Ph.D. student Lise Sohl Jeppesen, Department of Nutrition, Exercise and Sports, University of Copenhagen.

The school’s staff are responsible for the daily implementation of the activities.

On the following pages, we have described what the project is about and what we would like your child to participate in if you and your child would like to be part of the scientific study. Before you and your child decide to participate, please read the information on the following pages carefully to ensure that you understand what the project entails. We hope that your child will be part of the project. Please note that participation in the scientific study is voluntary. Even if you have decided to participate, you and your child can withdraw from the project at any time without giving any explanation as to why you no longer wish to participate.

In the attached participant information, you can read more about what the experiment entails, which methods are used, and your and your child's rights if you choose to say yes to participate in the research project.

On behalf of the Active School Team,

Anna Bugge

Associate professor, Ph.D., Cand.scient,

Department of Midwifery, Physiotherapy, Occupational Therapy and Psychomotor Therapy, University College Copenhagen, Copenhagen, Denmark

E-mail: [abug@kp.dk](mailto:abug@kp.dk)

**Purpose of the study**

We intend to investigate how physical activity can be best implemented in everyday school life and whether learning increases among 3^rd^ grade pupils who receive either ‘Move & Learn’ or ‘Run, jump & fun’ interventions.

No biological material will be collected as part of the study.

**The intervention**

All children in the participating classes will take part in one of two planned activities: 1) physically active learning during Danish class twice a week and math class twice a week (*Move & Learn)*; 2) physical activity outside of regular class four times a week (*Run, jump & fun)* or as a control class, which continues their school day unchanged. These activities will be led by the children´s own teacher/pedagogues.

**Plan for the scientific study**

Participation in the scientific study requires your consent for your child to participate. The study will take place at the school under safe and comfortable conditions, and all activities will be planned in close cooperation with school management and staff.

Measurements will be taken prior to and towards the end of the project.

Data collection for the scientific study will be conducted on one school day but will only require a small part of the day.

The testing consist of:

• The child will complete motivation and well-being questionnaires, which will be done individually in the classroom and all questions, will be read aloud.

• A computer-based cognitive test of attention and reaction time will be conducted. These measurements will take approximately 30-60 minutes.

• The child will complete a standardized Danish and math test.

• The fitness test will take place in the gym and will be a running test where children will run for 15 seconds and stand still for 15 seconds for 10 minutes. The children will run between two lines, and a test leader will guide them. The goal is to reach as far as possible, and it is not a competition against other children.

• Height, weight and waist circumference will be recorded in a private room, where the child will also complete a puberty questionnaire (self-determination of puberty development).

• Some children will be asked to wear an activity monitor for a week to measure their physical activity levels. The monitor should be worn all the time and not just during school hours.

• Some children will be invited to an interview about their experiences with more movement in the school day.

Children in the involved classes will take part in physical activity as part of their normal school day, except for children in control classes.
Data for the scientific investigation will only be collected for the children for whom informed consent has been given by the parents or guardians. When informed consent is not given, your child will off course take part in the physical activities just like all the other children.

**Side effects, risks, complications and inconvenience.**

The measurements mentioned consist solely of questionnaires and behavioral measurements without any risk of side effects. The fitness test may cause some discomfort (such as feeling out of breath and tired), but trained personnel present will be present to ensure your child feels safe. During the study period, your child will have an increased level of physical activity during the school day through activities related to the project, which involves a risk of falling or twisting an ankle, as with any other physical activity. This risk is, of course, also a part of regular physical education lessons or school breaks.

**Benefits of the scientific study**

It is well documented through a series of studies that regular physical activity is accompanied by a range of positive health effects, and there is good evidence that it can have positive effects on motor skills and cognitive functions. We hope, the project will contribute with important information on how physical activity can be appropriately implemented in schools, with the aim of engaging and motivating both students and teachers.

**Exclusion from and interruption of the study**

Your child will be excluded from the scientific study if we have not received informed consent for participation. There may be unforeseen technical problems with the equipment, which may require us to interrupt or postpone the experiment. However, in this case, your child will only be excluded from data collection in the scientific study and of course not from participation in the physical activities at school.
It is unlikely that incidental findings occur in the classical sense when using the described scientific methods. In the (rare) cases where incidental findings, like mis-thriving, which need further investigation may occur, the person responsible for the research will contact the school for further contact to you, as parents, and relevant authorities.

**Information on financial matters**

The Active School study is supported by the Independent Research Fund Denmark. The grant holder is Associate Professor Anna Bugge, Department of Midwifery, Physiotherapy, Occupational Therapy and Psychomotor Therapy, University College Copenhagen, who together with the Department of Nutrition, Exercise and Sports, University of Copenhagen also provides financial support the project.

**Access to the study’s results**

Consent for your child’s participation in the scientific part of the project will include processing of collected data.

All information that emerges from the collected data in the research project is confidential and can only be disclosed as part of the compulsory control by relevant authorities. During the collection of results these will be anonymized, so individual participants will only be identified by a participant ID number.

Only employees who are directly involved in the collection, analysis, and processing the results of the data will have access to the data. Both inconclusive, positive and/or negative findings will be attempted to be published. It may take several years before all results are published.

You will not have access to your child’s own data, but the participant group will receive a common information about the project´s general results. This is done through an oral joint briefing after the projects´ completion. There is also an opportunity to request ongoing published results from the project.

**Ethics and handling of data**

All data collected in the study will be stored on a secured data drive and treated confidentially and in accordance with rules from the Data Protection Authority and the General Data Protection Regulation (GDPR). All data will be anonymized or deleted.

At the publication of the results, all participants will be anonymous. It will be possible upon written request, to obtain insight into one´s own data. The study has been approved by the Local Scientific Ethical Committee, University of Copenhagen. Participation is voluntary and free, and it is possible to withdraw from the project anytime and without any explanation being required. However, participating in ’Run, jump & fun’ or ’Move & learn’ is part of the obligatory school day.
You and your child will not receive payment for participating in the project.
Further general information about trial participants´ rights can be found in the link below. We apologize that it is only published in Danish by the National Scientific Ethical Committee:

<https://nationaltcenterforetik.dk/Media/638090459963282027/Forsoegspersoners%20rettigheder%20NVK.docx>

You have the opportunity to obtain access to documents according to the rules of the Public Access to Information Act. If you as parents/guardians or your child - unexpectedly - were to be subjected to unsatisfactory treatment, you have the opportunity to complain. If you need this, we will provide further information and any relevant forms.

We hope that you would like to participate in the project! You are of course always welcome to contact us if you want further information or have any questions about the project.

**Summary**

We hope that this information sheets has given you sufficient insight into what it means to participate in a scientific study, and you therefore feel equipped to make a decision regarding your child’s participation.

If you would like to know more about the project and the scientific study, you are very welcome to contact the project coordinators, see contact details below:

Malene Stolpe Norup, associate professor and

Lise Sohl Jeppesen, phd-student
[activeschool@nexs.ku.dk](mailto:activeschool@nexs.ku.dk)

**2. Consent form for parents**

**Consent from the holder of parental authority to their child participation in a health science research project.**

**Title of the researchproject: "ACTIVE SCHOOL in a school year"**

**Statement from the holder of parental authority:**

- I/we have received written and oral information and I/we know enough about the purpose, method, advantages and disadvantages

**to provide my/our concent.**

- I/we know that participation is voluntary and that I/we can always withdraw my/our consent without my/our daughter/son losing their current or future rights to participate in the project or otherwise

Writhe the child´s name:

Write the child´s birth:

I/we provide consent for ________________

The child´s class:

School:

Participates in "ACTIVE SCHOOL in a school year".

Name or names of the parental authority(ies):_________________ _________________________

Below you will be asked to sign the consent regarding your child can participation in the ACTIVE SCHOOL project and that you have read/seen and understood the information.

You sign by clicking the "Add Signature" button.

The information can be seen in the video below and read at the very bottom of this consent form.

Information will be visible by marking the "Show more information" checkbox

Please watch the video below about the consent

By clicking the "watch video" button

Link <https://video.ku.dk/secret/86297243/2e2407a6082b3d06a23969b137b2f5ec>

I/we will get a copy of this consent form and the participation information for our own usage.

Date:

Signature:

Date:
Signature:

1. **Written information to teachers**

Participant information about ACTIVE SCHOOL for teachers, educators, and other adults employed at the school.

**Purpose of the study**

The purpose of Active School is to investigate the effect of two different interventions: "Move & Learn" or "Run, Jump & Fun"

**Intervention (mandatory for all participants in the project)**

All adult participants in the project (school staff) will continuously participate in preparatory and developmental activities to acquire knowledge and skills to work with the interventions. Additionally, meetings and courses will be conducted as agreed upon to maintain focus on the interventions throughout the year. Your school has entered into a written agreement regarding this. Furthermore, assistance will be provided in obtaining consent from parents of children participating in the project. A link to the parental consent form will be sent prior to the project start and can be shared via Aula or another platform. The school will be randomly assigned to one of the following three groups for the school year 2023-24:

Move & Learn: physical activity integrated into Danish lessons twice a week for 30 minutes each and into math lessons twice a week for 30 minutes each. As an instructor in this group, you will be responsible, together with your team, for implementing this.

Run, Jump & Fun: physical activity outside of regular subjects four times a week for 30 minutes each. As an instructor in this group, you will be responsible, together with your team, for implementing this.

Control group: in this group, you will continue your teaching and daily routine without any new interventions. Control schools will be offered courses in one of the two physical activity programs after the summer break in 2024.

Plan for implementation study for groups 1) Move & Learn and 2) Run, Jump & Fun

Your participation in the scientific study of the implementation itself requires your consent.

The implementation study will be conducted as a process evaluation, including the collection of materials for the scientific study before, during, and after the school year 2023-24.

• Adult participants will respond to a short SMS questionnaire every week (excluding holidays).

• Participants will complete an electronic questionnaire before and after the school year.

• Some participants will be invited for interviews regarding their experiences with the interventions.

• Some participants will be visited by researchers who will observe practices during the school year.

Plan for implementation study for group 3) control classes

Your participation in the scientific study of the implementation itself requires your consent.

To compare the activity levels of the two intervention groups, data will be collected on the control group's regular teaching during the school year 2023-24.

• Adult participants will respond to a short SMS questionnaire regarding physical activity in teaching every week (excluding holidays).

Plan for effectiveness study (all groups)

Another part of the study focuses on the effectiveness of the interventions on the students. The participating teachers and educators will assist researchers in assessing the students' fitness, physical activity levels, motivation, and well-being using tests and questionnaires at selected times.

Additionally, the participating Danish and mathematics teachers in the classes will conduct standardized tests twice during the 3rd grade, respectively.

**Benefits of the scientific study**

Numerous studies have provided evidence that regular physical activity is accompanied by several positive health effects. There is also good evidence that physical activity can have positive effects on motor skills and cognitive functions. Furthermore, successful school projects require a focus on the implementation of interventions.

The ACTIVE SCHOOL project will hopefully contribute valuable information on how physical activity can be effectively implemented in schools to enhance learning and engage and motivate both students and teachers.

**Information on financial matters**

The scientific study, as well as the physical activities and the project as a whole, is supported by funding from the Danish Independent Research Fund. The grant holder is Anna Bugge, Associate Professor at the Department of Therapy and Midwifery Education, Copenhagen University College, which also provides financial support to the project along with the Department of Nutrition and Exercise, University of Copenhagen.

**Access to research results**

Consenting to your participation in the scientific study includes the processing of necessary information related to the collected results. All information obtained in the project is confidential and can only be disclosed as part of the relevant authorities' legally required oversight of the scientific project. The collected results will be anonymized, with individual participants identified only by a participant number. Only the staff directly involved in collecting, analyzing, and compiling the conducted studies will have access to the data. Inconclusive, negative, and positive findings will be attempted to be published. It may take several years before all results are published.

You will not have access to your own data, but the participant group will receive collective information about the project's general results. This will be done through a joint briefing after the project's completion. Additionally, ongoing published results from the project can be requested.

**Ethics and data handling**

All information from the studies/measurements will be stored on a secure data drive and treated confidentially in accordance with the regulations of the Danish Data Protection Agency and GDPR. Anonymization and personal data will be deleted at the end of the project. When the results are published, all participants will remain anonymous. The project has been approved by the Scientific Ethics Committee. Participation in the project is voluntary and free, and it is possible to withdraw from the project at any time without stating a reason. However, it will not be possible to opt out of implementing teaching with the "Learn with the Body"/"Fun, Pulse & Power" interventions, as they are part of the written agreement with your school. Participation in the project does not include any remuneration. We encourage you to read "Subjects' Rights in a Biomedical Research Project found at <https://nationaltcenterforetik.dk/Media/638090459963282027/Forsoegspersoners%20rettigheder%20NVK.docx>

We apologize that it is only published in Danish by the National Scientific Ethical Committee:

We sincerely hope that you will benefit from participating in the project! You are always welcome to contact us if you require further information or have any questions.

You have the opportunity to request access to information under the rules of the Public Access to Information Act. If, contrary to expectations, you experience unsatisfactory treatment as a participant, you have the option to file a complaint. If you need assistance with this, we will provide you with additional information and any relevant forms.

**Summary**

We hope that this information has provided you with sufficient insight into what it means to participate in the scientific study and that you feel prepared to make a decision regarding your participation.

If you would like to learn more about the project and the scientific study, you are welcome to contact the project leaders using the contact details below:

Malene Norup, Associate Professor, Ph.D., Department of Therapy and Midwifery Education, Copenhagen University College. Email: activeschool@nexs.ku.dk or phone: 4189 8573.

Anna Bugge, Associate Professor, Ph.D., Department of Therapy and Midwifery Education, Copenhagen University College.

Jacob Wienecke, Associate Professor, Ph.D., Department of Nutrition and Exercise, University of Copenhagen.

Lise Sohl Jeppesen, Ph.D. student, Department of Nutrition and Exercise, University of Copenhagen.

1. **Consent Form teachers**

**Consent from adult participants (school staff) for participation in a scientific research study.**

**Name:**

**Participant's statement:**

**🞎 I give consent to participate in "ACTIVE SCHOOL 2023-2024."**

• I have received written information (attached), and I know enough about the purpose, method, benefits, and drawbacks to give my consent.

• I am aware that I have the right to withdraw from the study and revoke my consent at any time without losing my current or future rights to participate in the project or any other rights.

• I have been informed about how my data will be stored (sent in a letter).

**I have received a copy (receipt will be automatically sent via email) of this consent form and a copy of the written information about the project for my own use (confirmation of submission will also be automatically attached).**

**Date:**

**Electronic Signature:**

We also request additional information for use in the project, including recording demographic data of the participants (which will only be presented anonymously):

**Email address:**

**Provide an email address that you check weekly or more frequently.**

**Mobile:**

**Age:**

**Gender: (Male/female/other/do not want to answer)**

**Educational background:**

**Year of completion of education:**

**Number of years you have worked as a teacher/educator/other:**

**School:**

**How many classes are you a teacher/educator/other for, participating in Active School:**

1. **ACTIVE SCHOOL SPIRIT diagram displaying study recruitment, intervention and measures schedule.**

| **ACTIVE SCHOOL SPIRIT diagram displaying study recruitment, intervention and measures schedule.** | | | | | |
| --- | --- | --- | --- | --- | --- |
|  |  |  |  |  |  |
|  |  | **Study period** |  |  |  |
|  |  | Enrollment | Allocation | Post-allocation | Follow-up |
| **Timepoint** | | *Randomization* | *T0* | *Intervention* | *T1* |
| **Enrollment** | |  |  |  |  |
|  | *Written agreement with schools* | x |  |  |  |
|  | *Randomization into study arms* | x |  |  |  |
|  | *Baseline measures* |  | x |  |  |
| **Interventions** | |  |  |  |  |
|  | *Training of educators* | x |  |  |  |
|  | *Education of intervention teachers* |  | x |  |  |
|  | *Follow up - meetings for intervention teachers* |  |  | x |  |
|  | *Education of control teachers* |  |  |  | x |
| **Measures** | |  |  |  |  |
|  | *Participant characteristics (sex, birth date)* | x |  |  |  |
|  | *Cognitive tests* |  | x |  | x |
|  | *Academic tests* |  | x |  | x |
|  | *Physical activity measurement* |  | x |  | x |
|  | *Physical fitness test (Andersen)* |  | x |  | x |
|  | *SRQ-A questionnaire* |  | x |  | x |
|  | *Kidsscreen questionnaire* |  | x |  | x |
|  | *URP-I Survey* | x |  |  | x |
|  | *Process evaluation (RE-AIM)* |  |  | x | x |
|  |  |  |  |  |  |
